# Celebrating a decade of exercise physiology and metabolism research in physiological reports

**DOI:** 10.14814/phy2.15960

**Published:** 2024-02-23

**Authors:** David C. Wright, Arthur J. Cheng, Rebecca E. K. MacPherson

**Affiliations:** ^1^ School of Kinesiology University of British Columbia Vancouver British Columbia Canada; ^2^ Faculty of Land and Food Systems University of British Columbia Vancouver British Columbia Canada; ^3^ BC Children's Hospital Research Institute Vancouver British Columbia Canada; ^4^ School of Kinesiology and Health Science York University Toronto Ontario Canada; ^5^ Department of Health Sciences Brock University St. Catharines Ontario Canada; ^6^ Centre for Neuroscience St. Catharines Ontario Canada

**Keywords:** adipose tissue, exercise, metabolism, microbiome, muscle

## Abstract

During its first decade of life, Physiological Reports has become a home for well‐conceived and rigorously performed exercise physiology and metabolism studies. The breadth of research within this area is impressive, covering exercise‐induced increases in skeletal muscle gene expression to the effects of exercise on the gut microbiome. The purpose of the current review is to highlight some of the impactful exercise physiology and metabolism papers published in the journal and to look ahead to what areas exercise physiology publications might address in the next 10 years.

Physiological Reports is celebrating its 10th anniversary as a Gold Open Access Journal. Over the past decade, the journal has become a home for exercise physiology and metabolism focused papers that are well‐conceived, rigorously performed, and make important contributions to the field (Adams et al., 2023). The breadth of exercise and metabolism‐based papers published in the Physiological Reports is impressive, ranging from studies examining the impact of exercise on skeletal muscle gene expression (Ydfors et al., 2013), to the role of adipose tissue in the control of systemic glucose and lipid metabolism (Foster et al., 2013), and the effects of exercise on the gut microbiome (Taniguchi et al., 2018). The purpose of this review is to highlight some of the impactful exercise physiology and metabolism studies published in the journal over the past 10 years. The studies that are discussed were chosen as they were among the most highly cited papers relative to others published in the same year. This review also provides a roadmap of areas in exercise physiology and metabolism that are ripe for further exploration.

## 1 “MUSCLING IN” ON EXERCISE ADAPTATIONS

**FIGURE 1:**
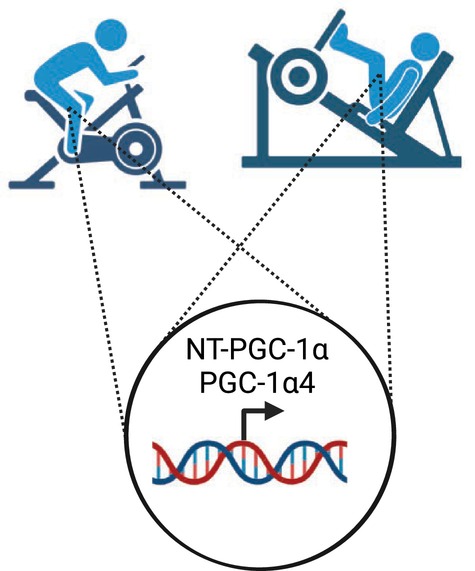
Aerobic and resistance exercise both induce the expression of NT‐PGC‐1α and PGC‐1α4 in human skeletal muscle. The figure was created using Biorender.

Skeletal muscle is a significant contributor to resting whole‐body energy expenditure (Zurlo et al., 1990), is quantitatively the most important organ for insulin mediated glucose disposal (DeFronzo et al., 1981), and is a crucial tissue involved in locomotion. Given these points, a large body of literature has focused on identifying the molecular signals driving exercise‐induced adaptations in skeletal muscle. A molecule that has received considerable attention is PPARγ co‐activator 1 alpha (PGC‐1α), a transcriptional co‐activator and regulator of mitochondrial biogenesis, autophagy, angiogenesis, and the induction of slow twitch muscle fibers (Halling et al., 2016; Igarashi et al., 2016; Martinez‐Redondo et al., 2015). There are a number of different splice variants of PGC‐1α including NT‐PGC‐1α and PGC‐1α4, and though these variants originate from different promoters, both encode shorter proteins which are thought to co‐activate different transcription factors than the full‐length protein. For instance, PGC‐1α4 is responsive to, and involved in, adaptations to resistance exercise such as muscle hypertrophy (Ruas et al., 2012) and this occurs independent of known PGC‐1α targets involved in aerobic exercise‐induced adaptations such as mitochondrial biogenesis and angiogenesis. Despite these purported differences in the function of PGC‐1α splice variants, Ydfors et al. (2013) found an equivalent induction of NT‐PGC‐1α and PGC‐1α4 following either an acute bout of aerobic or resistance exercise in human skeletal muscle (Figure 1). These findings are important as they suggest that the induction of truncated PGC‐1α splice variants does not appear to explain distinct adaptations that occur in skeletal muscle with resistance compared to endurance exercise.

Adaptations to exercise are governed not only by the mode, duration, and intensity of exercise itself but are also heavily influenced by postexercise nutrition (Wilkinson et al., 2023). For example, protein intake following resistance exercise is known to increase muscle protein synthesis. General recommendations for maximizing myofibrillar protein synthesis have typically recommended consuming ~20–25 g of protein following a bout of resistance exercise. However, these rough guidelines fail to consider if the total amount of lean body mass would impact the muscle protein synthetic response to protein ingestion following resistance exercise. To address this issue, Macnaughton et al. (2016) examined muscle protein synthesis in participants with lower or higher lean body mass following a session of whole‐body resistance exercise given 20 or 40 g of protein. While muscle protein synthesis was stimulated to a greater extent with the higher dose of protein, the synthetic response did not differ based on lean body mass. These findings provide novel insight that more protein is required than previously thought to maximize muscle protein synthesis when whole‐body resistance exercise is performed. Further research is required to determine the upper limit of ingested protein that can maximally stimulate muscle protein synthesis and if the amount of lean body mass impacts these responses.

## 2 THE REGULATION OF SYSTEMIC METABOLISM: MORE THAN JUST MUSCLE

**FIGURE 2:**
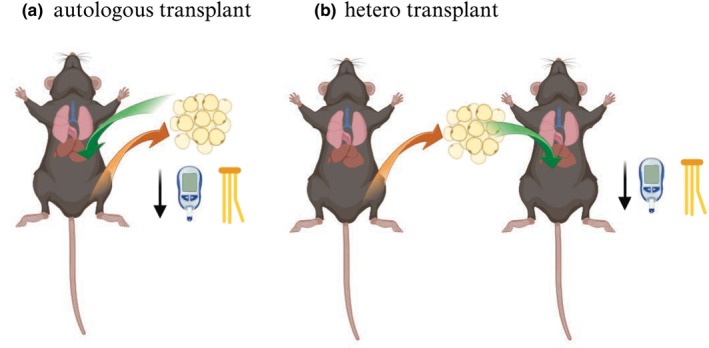
Subcutaneous fat transplantation within (autologous [a]) or between (hetero [b]) mice with diet‐induced obesity improves glucose tolerance and lipemia. The figure was created using Biorender.

White adipose tissue was once thought of as an inert storage depot of excess calories. However, it is now widely recognized as a critical regulator of whole‐body glucose and lipid metabolism. As eloquently described by da Silva Rosa and colleagues (2020), adipose tissue secretes a vast array of inflammatory cytokines and lipid moieties which can markedly impact skeletal muscle and liver insulin action. The metabolic effects of white adipose are not homogenous and vary according to the anatomical location of the depot with visceral, compared to subcutaneous adipose tissue being more closely associated with the development of insulin resistance, hypertension, and dyslipidemia. The important role of subcutaneous adipose tissue in the regulation of glucose and lipid metabolism was elegantly shown by Foster et al (2013) who demonstrated that autologous (excision and relocation of adipose within the same animal) or hetero‐transplantation (tissue from a donor) of subcutaneous adipose tissue into the visceral cavity of diet‐induced obese mice led to improvements in glucose homeostasis and reductions in portal triglyceride concentrations (Figure 2). This paper, using two distinct models of fat transplantation, provides causative evidence for beneficial metabolic effects of subcutaneous adipose tissue. Moving forward, it will be important to identify the factors secreted from adipose tissue, that is, adipokines and lipokines, that could be mediating these protective effects.

**FIGURE 3:**
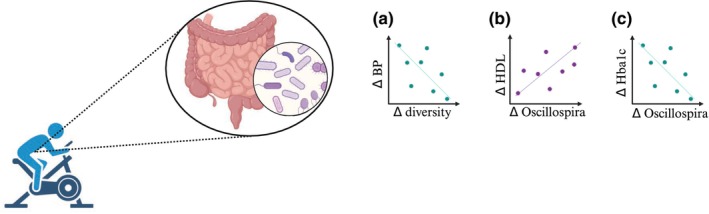
Changes in microbial diversity are negatively associated with blood pressure (a) while changes in *Oscillospira* are positively associated with changes in HDL cholesterol (b) and negatively associated with changes in HbA1c (c) in elderly male participants following exercise training. The figure was created using Biorender.

In addition to adipose tissue, there is growing evidence suggesting that the gut microbiome is an important contributor to overall health and is responsive to exercise (Zheng et al., 2022). While in human participants it is difficult to establish causation between alterations in the gut microbiota and cardiometabolic health, exploring associations between individual bacterial species or microbial diversity with indices of cardiovascular and metabolic health can provide insight into potentially important relationships. Taniguichi and colleagues (2018) examined changes in gut microbiota with endurance exercise training in a cohort of elderly Japanese men and found a negative correlation between changes in microbial diversity and blood pressure. When examining specific bacterial species, they further discovered a positive correlation between changes in the abundance of *Oscillospira* and HDL cholesterol and a negative correlation between *Oscillospira* and HbA1c following exercise training (Figure 3). These findings highlight the plasticity of the gut microbiome to exercise and suggest the involvement of the gut in perhaps mediating a portion, of the metabolic effects of exercise. These findings set the stage for further research aimed at elucidating how changes in the gut microbiome could be linked to cardiometabolic disease risk and if the observed changes reported by Taniguichi and associates (2018) are observed in other populations such as women and those with obesity and cardiometabolic disease.

## 3 WHERE WILL THE NEXT 10 YEARS TAKE US?

In its first decade of life, Physiological Reports has served an important role for the physiology community as a venue for well‐conceived and executed exercise and metabolism focused investigations. In the coming years, studies examining interactions between exercise and nutritional and/or pharmacological interventions in preclinical and patient populations (lean, with obesity, diabetes, cardiovascular disease etc.) and across different stages of life (children, elderly) are highly encouraged.

With the increasing popularity of “omics”‐based platforms, hypothesis generating approaches could provide important new clues into the mechanisms underlying the effects of exercise on numerous tissues. Extending beyond tissue‐based analysis, the advent of single‐cell omics allows for increasing precision in elucidating the effects of exercise in heterogenous tissues such as skeletal muscle and adipose tissue.

Lastly, wearable biometric devices can now provide a wide range of real time cardiovascular, metabolic, and performance‐based data in a real‐world setting allowing researchers to move exercise intervention studies outside of the laboratory. Further embracing these technologies will allow for better translation of research findings. As always, authors should continue to consider sex and gender in the design of their studies.

## FUNDING INFORMATION

No funding was received for this work.

## CONFLICT OF INTEREST STATEMENT

The authors have no conflict of interest to declare.

## ETHICS STATEMENT

DCW is the Deputy Editor and AJC and REKM are Editorial Board members of Physiological Reports. The authors were blinded from reviewing or making decisions for the manuscript.
